# The efficacy of serratus posterior superior intercostal plane block performed under ultrasound guidance in thoracic postherpetic neuralgia: a retrospective cohort study

**DOI:** 10.1186/s12871-026-03817-9

**Published:** 2026-04-21

**Authors:** Halil Ibrahim Altun, Gozde Altun

**Affiliations:** 1 Department of Anesthesiology and Reanimation, Division of Pain Medicine, Istanbul Kanuni Sultan Suleyman Training and Research Hospital, Atakent Mh, Turgut Ozal Bulvari No:46/1, Istanbul, 34303 Türkiye; 2 Department of Anesthesiology and Reanimation, Istanbul University-Cerrahpasa, Institute of Cardiology, Haseki Caddesi No:32, 34096, Fatih, Cerrahpaşa Mah, Org. Abdurrahman Nafiz Gürman Cd. Bina no.24, 34098, İstanbul, Türkiye

**Keywords:** Postherpetic neuralgia, Serratus posterior superior intercostal plane block, Neuropathic pain, Regional block

## Abstract

**Background:**

Postherpetic neuralgia (PHN) is a neuropathic pain syndrome that can be resistant to traditional therapies and severely impairs quality of life. The serratus posterior superior intercostal plane block (SPSIPB) is a recently described regional anesthesia technique conducted under ultrasound guidance, thought to provide analgesia for thoracic neuropathic pain. This research analyzed the effectiveness and safety of SPSIPB in patients with thoracic PHN.

**Methods:**

Thirty-four thoracic PHN patients who underwent SPSIPB between June 2023 and February 2025 were retrospectively evaluated. Pain intensity was evaluated utilizing the Numerical Rating Scale (NRS-11), neuropathic pain symptoms using the Douleur Neuropathique 4 (DN4) questionnaire, and quality of life using the 12-item Short Form Health Survey (SF-12). Measurements were taken before the procedure and at 1 month and 3 months post-procedure.

**Results:**

Thirty-four patients were included. Mean patient age was 68.4 ± 7.1. Among all patients, 50% were female. The NRS-11 scores decreased from 7.6 ± 1.1 before the procedure to 2.8 ± 1.3 at 3 months (*p* < 0.001). The DN4 scores also decreased from 6.4 ± 1.0 before the procedure to 3.1 ± 1.2 at 3 months after the procedure (*p* < 0.001). Significant increases were observed in both the physical and mental component scores of the SF-12 (*p* < 0.001). No severe complications were encountered.

**Conclusion:**

SPSIPB administered under ultrasound guidance is associated with a reduction in pain intensity and an improvement in quality of life in patients with thoracic postherpetic neuralgia. However, given the study’s retrospective design and the absence of a control group, these outcomes should not be considered indicative of a causal effect; instead, they should be interpreted as hypothesis-generating. Prospective, randomized controlled trials are needed to demonstrate the efficacy of SPSIPB and its comparative benefits relative to other interventions.

**Trial registration:**

This retrospective single-center study was conducted in accordance with the Helsinki Declaration. It was registered on clinicaltrials.gov (NCT07067892).

## Introduction

Postherpetic neuralgia (PHN) is a chronic pain syndrome that can be resistant to treatment [1]. It may lead to physical and social disability and psychological disorders, and last for years. The varicella zoster virus remains latent in sensory neurons of individuals who have had chickenpox; reactivation of the virus results in herpes zoster (HZ). The HZ usually begins with dermatomal pain in most patients, and dermatomal rashes develop within a few days. These skin rashes heal within approximately 2–4 weeks. However, pain may persist even after the rash has healed [2]. The development of PHN occurs due to increased sensitivity in central neurons, decreased inhibitory neuron function, peripheral nerve inflammation and damage, and abnormal conduction resulting from this damage. The PHN is the most common complication of HZ, occurring in 9–19% of all patients who experience HZ. Its prevalence increases with age: approximately 2% in patients under 50, approximately 20% in patients over 50, and approximately 35% in patients over 80 [1–3]. Various treatment options, including medications and interventional therapies, are available for pain management in PHN. Systemic agents include tricyclic antidepressants, calcium channel alpha-2-delta blockers, serotonin-norepinephrine reuptake inhibitors, and opioids [4]. Lidocaine and capsaicin can be used topically. On the other hand, interventional treatment options for PHN include epidural and intrathecal injections, sympathetic nerve blocks, plexus blocks, subcutaneous injections, and spinal cord stimulation (SCS) [5].

Thoracic epidural injections and sympathetic blocks used for thoracic PHN are technically challenging owing to anatomical constraints and may lead to serious complications, including pneumothorax and spinal cord injury [6, 7, 28]. By contrast, ultrasound-guided thoracic interfascial plane blocks are easier to perform, more superficial, and patients are not exposed to radiation. Since these blocks are performed with real-time ultrasound visualization, risks of vascular or neural injury and pneumothorax are further minimized [8, 29, 30]. The serratus posterior superior intercostal plane block (SPSIPB) is a newly described plane block that has recently been adopted in both anesthesiology and pain medicine practice. The serratus posterior superior muscle is located deep to the rhomboid major and minor muscles. The ligamentum nuchae originates from the spinous processes of the C7-T3 vertebrae and terminates at the upper border of the costal angle of the 2nd-5th ribs. The SPSIPB provides sensory block in the C3-T10 dermatomal distribution [9]. The SPSIPB has a wide dermatomal distribution when applied with ultrasound (USG) guidance.

The primary objective of this study was to assess changes in pain among patients with PHN who underwent SPSIPB, as measured by the NRS-11. Secondary objectives included evaluating the effect of SPSIPB on neuropathic pain characteristics using the DN-4 scale and examining its impact on quality of life using the SF-12 scale.

## Materials and methods

Adult (age > 18) patients who underwent a single-session SPSIPB in the thoracic region under USG guidance for PHN between June 2023 and February 2025 in our center constituted the target population of our study. After obtaining ethical committee approval (Health Sciences University, Istanbul Kanuni Sultan Süleyman Training and Research Hospital, KAEK/2025.05.139), electronic patient folders were retrospectively reviewed. Patients with cervical disc herniation, history of trauma or surgery to the neck, shoulder, or back, history of malignancy, kyphoscoliosis, inflammatory disease (rheumatoid arthritis, ankylosing spondylitis), congenital anomalies of the spine, neck pain accompanied by neurological deficits, pregnancy, mental and psychotic disorders, hematological diseases causing bleeding and coagulation disorders, use of antiplatelet, anticoagulant medications, systemic and local infection in the intervention area, allergy to any of the medications to be used, and patients who underwent an intervention for PHN were excluded. The SPSIPB was applied to patients with complaints (Numeric Rating Scale-11 > 4) persisting for more than 3 months despite medical treatment.

This retrospective single-center study was conducted in accordance with the Helsinki Declaration. It was registered on clinicaltrials.gov (NCT07067892) during data collection. Written informed consent was obtained from all patients. The study was designed in accordance with the STROBE (Strengthening the Reporting of Observational Studies in Epidemiology) guidelines [10].

### Data collection and assessment scales

The following data were collected: Age, gender, body mass index (BMI), affected side in PHN, duration of PHN and affected dermatome, history of HZ vaccination (live/recombinant), data regarding immunosuppression (Human Immunodeficiency Virus, active cancer, high-dose steroids), PHN family history, level of procedure performed, complications, Numeric Rating Scale-11 (NRS-11), Neuropathic Pain Questionnaire (DN4), Short Form-12 (SF-12) quality of life index subscores and the daily average tramadol consumption (mg/day) before and 1 and 3 months after the procedure were recorded.

#### Numeric rating scale − 11 (NRS-11)

NRS-11 is an 11-point numerical scale that allows patients to rate their pain from 0 (no pain) to 10 (the most severe pain they have ever experienced) [11]. Patients with NRS > 4 were included in the procedure.

#### Neuropathic pain questionnaire (DN4)

It is a practical screening tool developed by the French Neuropathic Pain Group to detect neuropathic pain. The scale has been widely used since 2005. It is a questionnaire consisting of 4 questions and 10 sub-items. Seven items related to pain quality (i.e., sensory and pain descriptors) are based on a patient interview. These components refer to how the pain feels to the patient. Three items are based on clinical examination. The clinician assesses whether the sensation of touch or needle prick is reduced (hypoesthesia) and whether light brushing increases or causes pain (allodynia). In this test, which is scored out of 10 points, a score of 4 or higher indicates neuropathic pain [12].

#### Short form 12-item health survey (SF-12)

The SF-12 consists of 8 subscales and 12 items: physical functioning (2 items), physical role (2 items), bodily pain (1 item), general health (1 item), energy (1 item), social functioning (1 item), emotional role (2 items), and mental health (2 items). Items related to physical and emotional roles are answered as yes or no, while other items have Likert-type options ranging from 3 to 6. The Physical Component Scale-12 (PCS-12) score is derived from the general health, physical functioning, physical role, and body pain subscales. In contrast, the Mental Component Scale-12 (MCS-12) score is derived from the subscales of social functioning, emotional role, mental health, and energy. Both the PCS-12 and MCS-12 scores range from 0 to 100, with higher scores representing better health [13].

#### Concomitant medications and analgesic adjustment

Patients maintained their existing pharmacological treatments for PHN, including gabapentinoids, amitriptyline, and duloxetine, at unchanged dosages. These regimens were not standardized across participants, reflecting real-world clinical practice.

Tramadol dosages were adjusted as needed following SPSIPB to optimize pain control. Mean daily tramadol consumption was recorded prior to the procedure and at 1 and 3 months post-procedure.

### Outcomes

The primary outcome is changes in pain intensity, as measured by the NRS-11, at pre-procedure, 1-month, and 3-month assessments in patients with thoracic postherpetic neuralgia who underwent SPSIPB. Secondary outcomes included changes in DN4 scores, which measure neuropathic pain components, and in SF-12 PCS-12 and MCS-12 scores, reflecting patients’ physical and mental quality of life. These measures were assessed at the same time points in order to examine the effects of SPSIPB on neuropathic pain characteristics and quality of life.

### Procedure

**Fig. 1:**
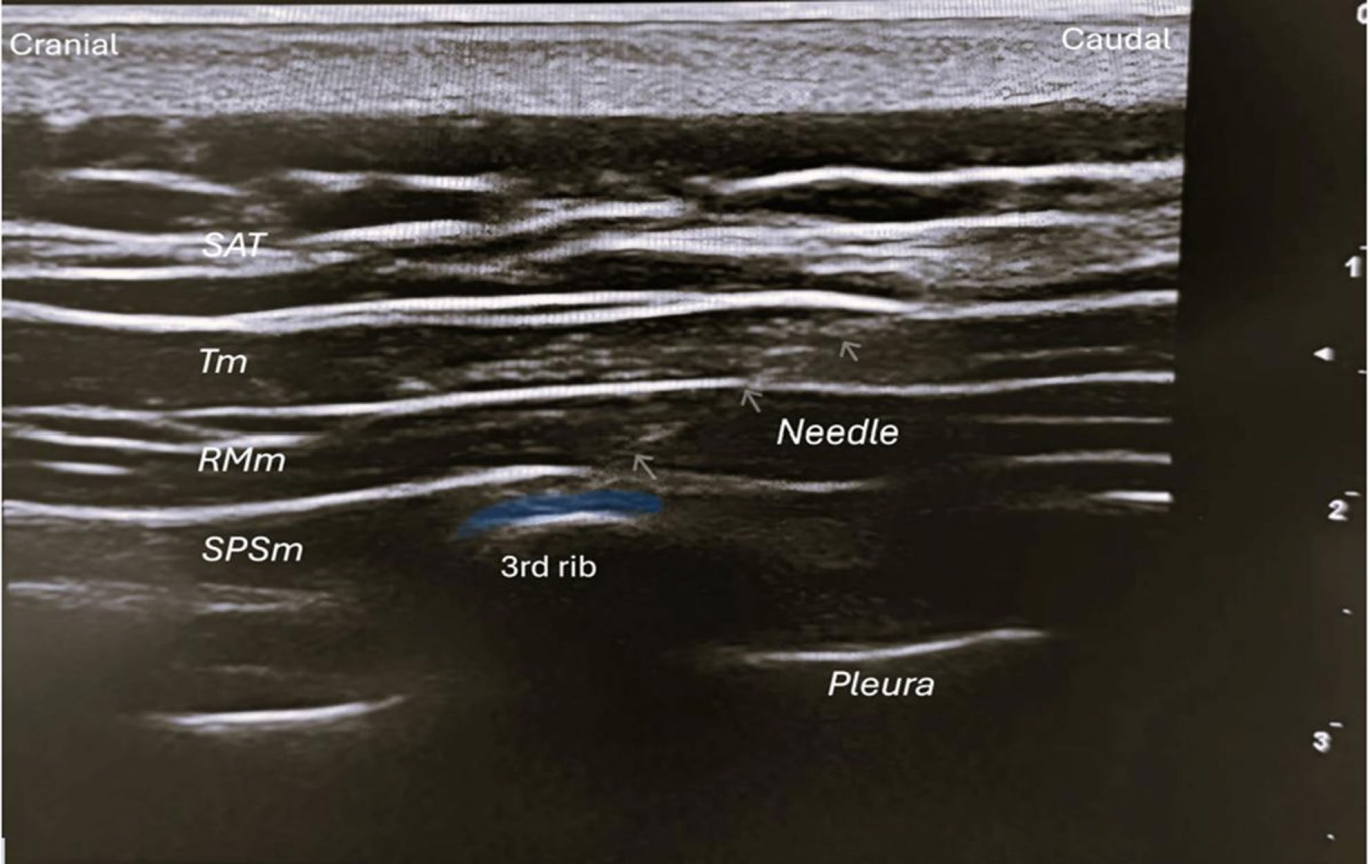
Serratus posterior superior intercostal plane block (SPSIPB). SAT: Subcutaneous adipose tissue, Tm: Trapezius muscle, RMm: Rhomboid major muscle SPSm: Serratus posterior superior muscle. The blue-shaded area indicates the intended target injection plane for the SPSIPB, with the needle advanced in a caudal-to-cranial direction under linear ultrasound probe guidance

After standard monitoring (blood pressure, electrocardiogram, saturation), a venous access was established in the forearm. All procedures were performed by the same pain physician with more than 5 years of experience with ultrasound. The patient was placed in the prone position, prepped with sterile povidone-iodine, and draped under sterile conditions. A pillow was placed under the chest to ensure optimal visualization. Under USG guidance, an 8 cm 22-Gauge peripheral nerve block needle was used in-plane at the level of the superomedial border of the scapula using a 7–12 MHz linear probe (Esaote, Mylab X7, Genova, Italy). The needle was directed caudal to cranial. The position was confirmed by injecting 1 ml of saline. An SPSIPB was performed at the T3 level. During the block procedure, a total of 30 ml of drug mixture was used: 0.25% bupivacaine/28 ml + 8 mg/2 ml dexamethasone (Fig. 1). After the procedure, patients were monitored in the day care unit for at least 2 hours. Patients without additional problems were discharged. No complications were observed during the procedure or during patient follow-up with the single-session SPSIPB.

### Statistical analysis

Data were analyzed using IBM SPSS Statistics Standard Concurrent User Version 26 (IBM Corp., Armonk, New York, USA) statistical software package. Descriptive statistics were presented as numbers (n), percentages (%), mean±standard deviation, median, minimum, and maximum. The normality of the data distribution was assessed using the Shapiro-Wilk test, and the homogeneity of variances was evaluated using Levene’s test. The Friedman test was performed to examine the median NRS-11, SF-12, and DN-4 scores during the follow-up period. Significance values were adjusted using the Bonferroni correction for multiple tests. Statistical significance was set at *p* < 0.05. Due to the failure of variables from repeated measurements to meet the assumption of normal distribution at one or more time points, comparisons over time were performed using the Friedman test. Accordingly, median (minimum–maximum) values were used as the primary summary measure in comparative assessments. Mean ± standard deviation values were reported only as descriptive statistics to increase clinical interpretability. The effect size accompanying the Friedman test results was calculated using Kendall’s W, an appropriate measure, and the level of effect size (small, medium, large) was interpreted according to the threshold values recommended in the literature.

#### Sample size and power considerations

Although this study was designed as an exploratory pilot trial, we performed an approximate a priori sample size calculation using G*Power software (version 3.1.9.7, Heinrich-Heine-University Düsseldorf, Germany). For the primary endpoint (change in NRS-11 pain scores over time), we modelled a repeated-measures ANOVA with one within-subject factor (time, 3 levels). Assuming a medium effect size (f = 0.25), a two-sided α error of 0.05, a power (1–β) of 0.80, a correlation of 0.50 among repeated measurements, and a non-sphericity correction ε = 1, the required total sample size was 28 patients. To account for potential dropouts and the slightly lower efficiency of the corresponding non-parametric Friedman test, the target sample size was inflated by approximately 10% (to at least 31 patients). Ultimately, 34 patients were enrolled, providing a power clearly above the planned 80% [14, 15].

## Results

**Fig. 2:**
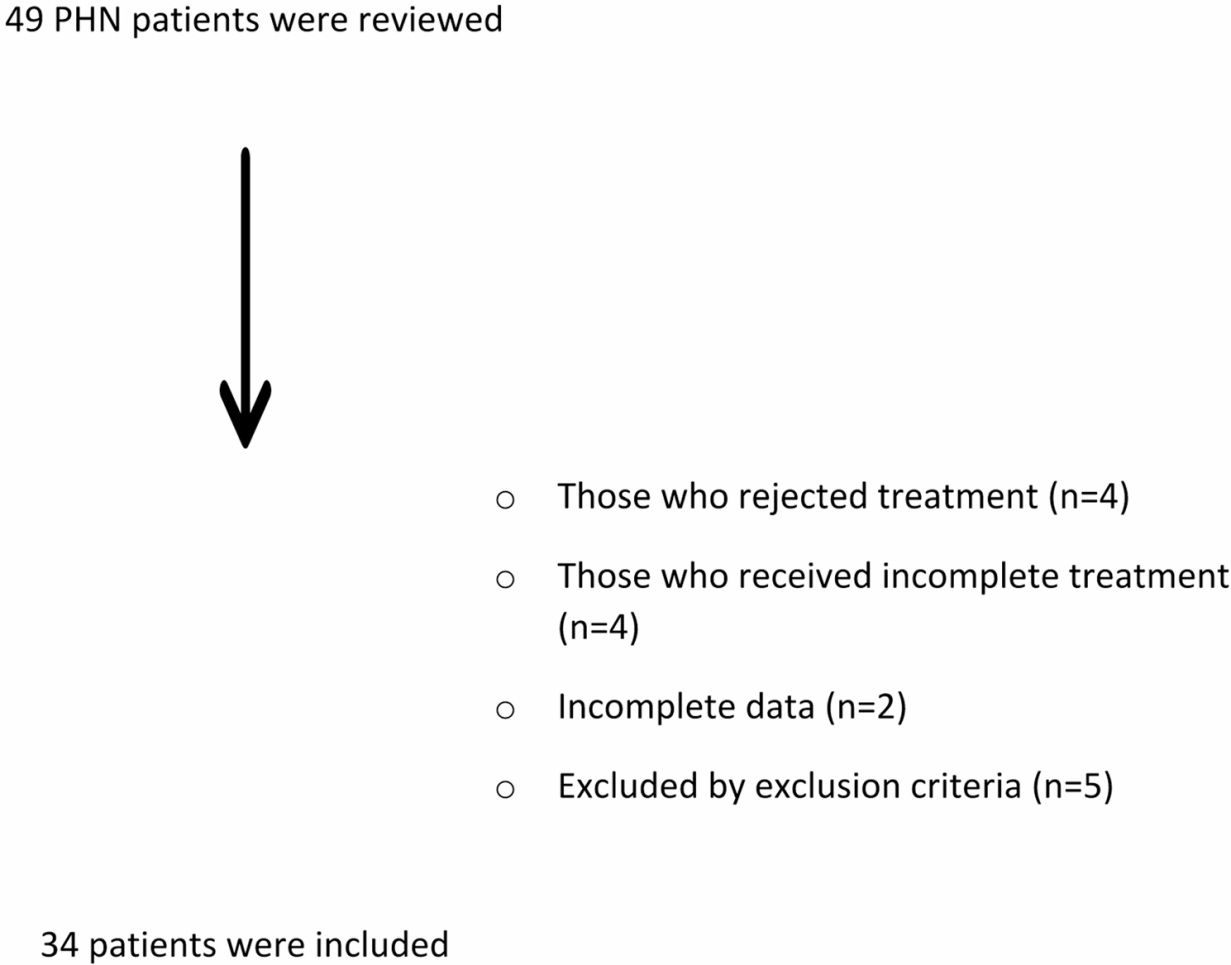
Flow chart (PHN: Postherpetic neuralgia)

A total of 49 patients presenting with PHN were identified via hospital records. Of these, 4 patients declined treatment, 4 received incomplete treatment, 2 had incomplete data, and 5 met the exclusion criteria. After applying the exclusion criteria, the data of the remaining 34 patients were analyzed (Fig. 2). There was an equal distribution of females (*n* = 17, 50%) and males (*n* = 17, 50%). The mean age of the participants was 69.47 ± 12.24 (95% CI: 65.2–73.7) years. In our study, cancer-related immunosuppression was present in 1 patient (3.4%). Due to the limited number of patients, we do not have sufficient data to draw a definitive conclusion. The symptom duration of the patients was 18.21 ± 14.19 months. In our study, none of the patients had received the HZV vaccine (live or recombinant). The demographic characteristics are presented in Table 1.

**Table 1:** Demographic features

Gender, *n* (%)	
Female	17 (50)
Male	17 (50)
Side of pain, *n* (%)	
Right	15 (44.12)
Left	19 (55.88)
Age, (*years*)	69.47 ± 12.24 73 (34–90)
BMI, (*kg*/*m*^2^)	28.51 ± 3.77 28 (21–36)
Duration of pain, *(months)*	18.21 ± 14.19 12 (4–60)
Immunsupression	1 (3.4)

*n:* Number of patients, Numerical variables are presented as mean±standard deviation or median (minimum-maximum) values

*BMI *Body-mass index

**Fig. 3:**
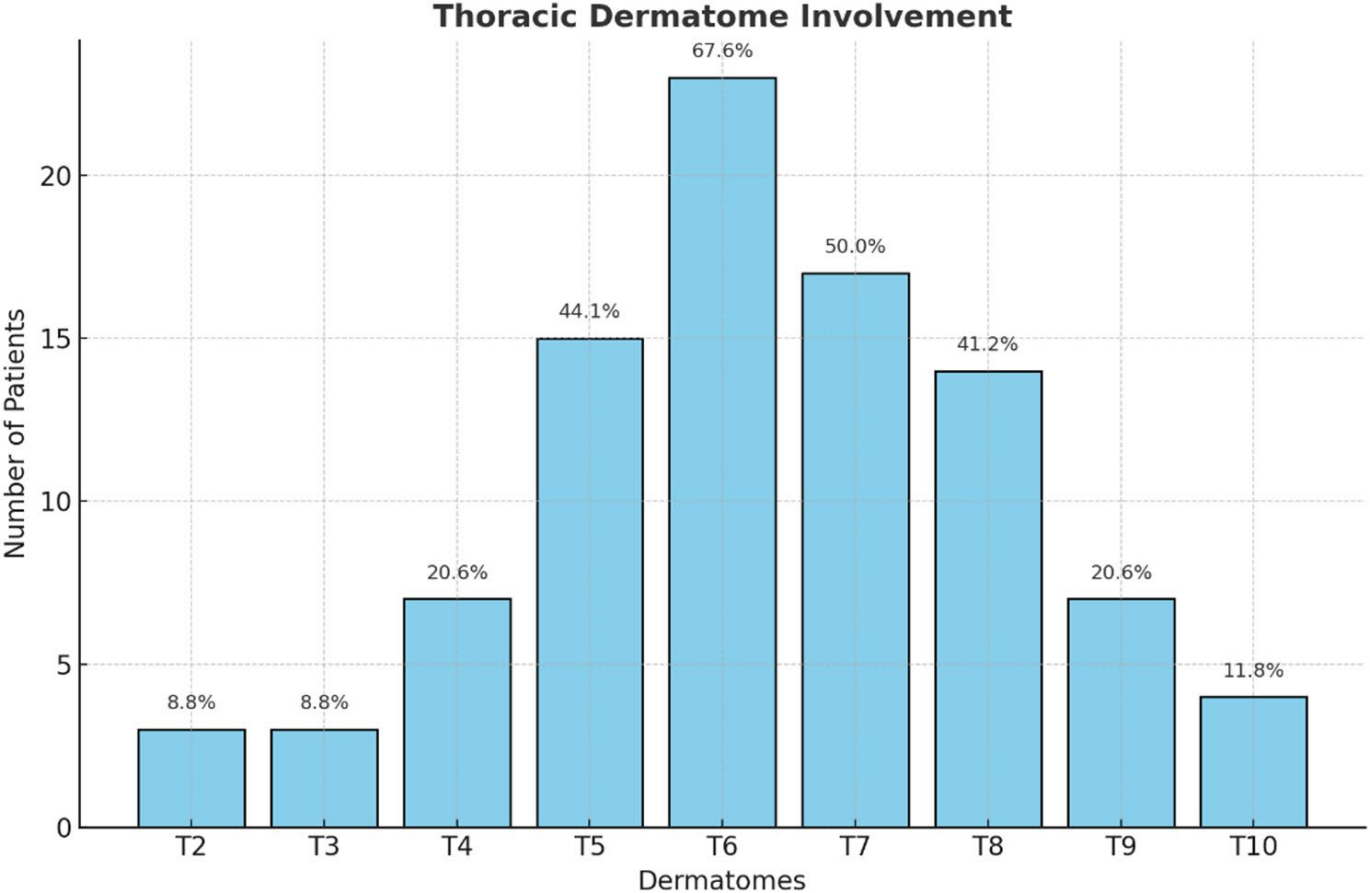
Affected thoracic dermatomes and prevalence of involvement in patients with postherpetic neuralgia. Percentages were calculated using the total study population (*n* = 34); cumulative percentages exceed 100% due to multiple dermatomal involvement in some patients

Thoracic dermatome involvement demonstrated a heterogeneous distribution. The T6 dermatome was most commonly affected (*n* = 23, 67.6%), followed by T7 (*n* = 17, 50%), T5 (*n* = 15, 44.1%), and T8 (*n* = 14, 41.2%). Less frequent involvement was noted in T4 (*n* = 7, 20.6%) and T9 (*n* = 7, 20.6%), T10 (*n* = 4, 11.8%), and in the upper thoracic dermatomes T2 (*n* = 3, 8.8%) and T3 (*n* = 3, 8.8%) (Fig. 3).

**Fig. 4:**
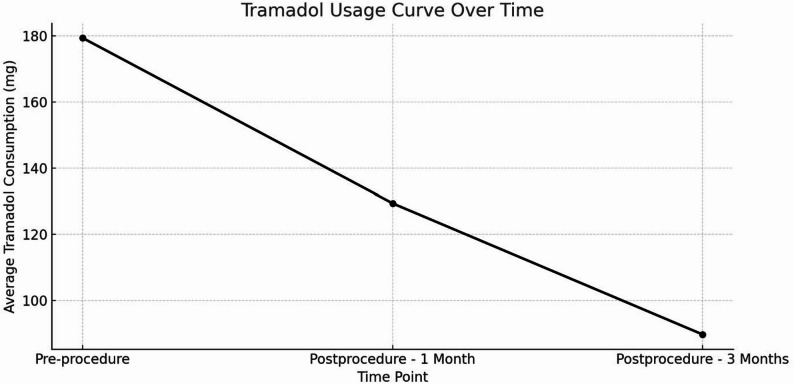
Mean daily tramadol consumption over time

Mean tramadol consumption also declined substantially over time following SPSIPB (Fig. 4). Pre-procedure tramadol use was 179.41 ± 59.18 mg (95% CI 158.76–200.06) and decreased to 129.4 ± 62.91 mg (95% CI 107.46–151.36) at 1 month and 89.71 ± 33.05 mg (95% CI 78.52–100.88) at 3 months. The Friedman test indicated a highly significant overall time effect (χ² (2) = 43.46, *p* < 0.001), with a large within-subject effect size (Kendall’s W = 0.639).

Considerable improvements in NRS-11, SF-12, and DN-4 scores were observed at 1 and 3 months compared with pre-procedure (*p* < 0.001). However, subgroup analysis found no statistically significant differences between the 1- and 3-month scores for NRS-11, SF-12, and DN-4 (Table 2).

**Table 2:** Comparative analysis of the pre-procedure, post-procedure 1st and 3rd month NRS, SF-12 PCS-12 and MCS-12 and DN-4 scores

		Mean (S.D.)	Median (min.-max.)	Test value*	*p*
NRS-11	Pre-procedure	8.24 (0.99)	8 (6–10)^a^	52.797	< 0.001
	1st -month	3.18 (1.57)	3 (1–6)^b^		
	3rd -month	3.44 (1.81)	3 (1–8)^bc^		
PCS-12	Pre-procedure	23.65 (4.17)	23 (14–34)^a^	53.368	< 0.001
	1st -month	44.03 (5.25)	44.5 (32–52)^b^		
	3rd -month	41.06 (7.83)	42 (22–53)^bc^		
MCS-12	Pre-procedure	23.76 (3.79)	24 (17–35)^a^	52.299	< 0.001
	1st -month	44.09 (5.73)	46 (31–52)^b^		
	3rd -month	41.97 (7.23)	44 (24–51)^bc^		
DN-4	Pre-procedure	7.76 (1.21)	8 (5–10)^a^	51.710	< 0.001
	1st -month	4.09 (1.42)	4 (1–6)^b^		
	3rd -month	4.26 (1.37)	4 (1–6)^bc^		

*Friedman test statistic, a-c: Values with the same superscript letter within each column do not differ significantly

*DN-4* Douleur Neuropathique 4, *NRS-11* Numerical Rating Scale-11, *PCS-12* Physical Component Scale-12, *MCS-12* Mental Component Scale-12

Mean NRS-11 scores decreased markedly over time following SPSIPB. Pre-procedure pain intensity was 8.24 ± 0.99 (95% CI 7.89–8.58) and fell to 3.18 ± 1.57 (95% CI 2.63–3.72) at 1 month and 3.44 ± 1.81 (95% CI 2.81–4.07) at 3 months (Table 2). The Friedman test demonstrated a highly significant overall time effect (χ² (2) = 52.8, *p* < 0.001), with a large within-subject effect size (Kendall’s W = 0.776).

Mean DN4 scores also decreased substantially over time. Pre-procedure DN4 was 7.76 ± 1.21 (95% CI 7.34–8.19) and decreased to 4.09 ± 1.42 (95% CI 3.59–4.58) at 1 month and 4.26 ± 1.37 (95% CI 3.73–4.68) at 3 months (Table 2). The Friedman test showed a highly significant overall time effect (χ² (2) = 51.7, *p* < 0.001), with a large within-subject effect size (Kendall’s W = 0.76).

Mean SF-12 scores increased markedly after the intervention. Pre-procedure MCS-12 was 23.76 ± 3.79 (95% CI 22.44–25.09) and rose to 44.09 ± 5.73 (95% CI 42.09–46.09) at 1 month and 41.97 ± 7.23 (95% CI 39.45–44.49) at 3 months. The Friedman test showed a highly significant overall time effect (χ² (2) = 52.30, *p* < 0.001), with a large within-subject effect size (Kendall’s W = 0.769).

Mean PCS-12 scores increased substantially after SPSIPB. Pre-procedure PCS-12 was 23.65 ± 4.17 (95% CI 22.19–25.10) and increased to 44.03 ± 5.25 (95% CI 42.20–45.86) at 1 month and 41.06 ± 7.84 (95% CI 38.33–43.79) at 3 months. The Friedman test indicated a highly significant overall time effect (χ² (2) = 53.4, *p* < 0.001), with a large within-subject effect size (Kendall’s W ≈ 0.785).

In an exploratory multiple linear regression model, the percentage reduction in NRS-11 pain from pre-procedure to 3 months was used as the dependent variable, and age, gender, duration of symptoms, pre-procedural daily tramadol dose, and BMI were entered as covariates. The overall model was not statistically significant (*R* = 0.35, R² = 0.13, adjusted R² = −0.03, F [5, 28] = 0.80, *p* = 0.557), indicating that these pre-procedural variables explained very little of the variance in the analgesic response. Longer symptom duration showed a weak, non-significant trend toward a smaller percentage reduction in pain (B = − 0.53% per month, 95% CI − 1.15 to 0.09, *p* = 0.089). In contrast, age, gender, pre-procedural tramadol dose, and BMI were not associated with the change in pain (all *p* > 0.65). No relevant multicollinearity was detected (all variance inflation factors < 1.2).

## Discussion

PHN is a disease that impairs quality of life and increases in frequency with age. Controlling pain in the early stages of PHN is essential. In acute herpes zoster (AHZ), pain and other symptoms subside and disappear within 1 month in most patients. The condition that persists beyond this period, defined as neuropathic pain lasting longer than 3 months, is termed PHN [16]. To our knowledge, there is limited direct evidence evaluating the efficacy of SPSIPB in thoracic PHN. However, the emerging literature suggests that fascial plane blocks (FPBs), including techniques associated with SPSIPB, may play a role in herpes zoster-related thoracic pain.

The incidence of PHN increases in patients with risk factors. Advanced age, immunosuppression, initial pain intensity, duration, presence of allodynia and hypoesthesia, pain interfering with daily activities, severe rash and prolonged rash duration, and ophthalmic localization are considered risk factors for zona [17]. Data regarding gender is conflicting. Some studies indicate that the incidence of PHN is lower in women, whereas others indicate that it is higher [18, 19]. In our study, 17 PHN patients were male (50%), and 17 were female (50%), indicating an equal incidence of PHN in both genders.

Hicks and colleagues reported an increased risk in patients with a family history of PHN [20]. In our study, 1 patient (3.4%) had a family history. Helgason and colleagues reported that patients under 50 years of age did not describe severe pain (i.e., VAS > 7), while the number of patients describing severe pain increased in patients over 60 years of age [21]. In our study, the mean patient age was 69 for women and 73 for men, while the mean NRS-11 score before the procedure was 8.24 ± 0.99 (95% CI 7.89–8.58). Consistent with Helgason et al.‘s study, our study found that patients’ ages and NRS-11 scores were relatively high. There was a significant decrease in NRS-11 after SPSIPB. (Kendall’s W = 0.776). The magnitude of the pain reduction observed after a single SPSIPB application appears to be greater than typically expected for chronic PHN. This may reflect reversion to the mean, placebo effects, concurrent pharmacological treatments, and the possible contribution of the dexamethasone adjuvant. The long-term effect is unknown due to the short follow-up period. Therefore, the results should be interpreted with caution.

In our study, since the data did not show a normal distribution, changes over time were evaluated using the Friedman test, and median values were used for comparisons. Mean ± standard deviation values were presented descriptively to increase the interpretability of Kendall’s W effect sizes, which indicate that the measurements demonstrated consistent changes over time, with moderate to high effect sizes. These results suggest that the findings are both clinically and statistically significant.

However, given the study’s retrospective, uncontrolled design, these outcomes should not be interpreted as evidence of causality and should instead be regarded as hypothesis-generating.

Immunosuppression is a defined risk factor for PHN [21]. In our study, cancer-related immunosuppression was present in 1 patient (3.4%). Due to the limited number of patients, we do not have sufficient data to draw a definitive conclusion.

In their review, Forbes et al. stated that pain limiting daily life activities in AHZ was a risk factor for PHN. In the study by Katz et al., accompanying biopsychosocial factors were investigated. These authors reported that a 1-unit increase in the Short Pain Inventory total score was associated with an 18% increased risk of PHN [17, 22].

Van et al. reported restrictions in daily living activities, sleep, physical functioning, and hobby maintenance in PHN patients [23]. In our study, patients’ physical, emotional, social, and mental health were assessed using the SF-12. The patients’ PCS-12 and MCS-12 scores were 23.65 ± 4.17 (95% CI 22.19–25.10) and 23.76 ± 3.79 (95% CI 22.44–25.09) at pre-procedure, respectively, and increased to 41.06 ± 7.84 (95% CI 38.33–43.79) and 41.97 ± 7.23 (95% CI 39.45–44.49) respectively, at the end of the third month after the SPSIPB.

Allodynia, paresthesia, hypoesthesia, burning, and itching are frequently associated with PHN due to involvement of sensory nerves. Antiepileptics and tricyclic antidepressants are preferred in the treatment of neuropathic pain in PHN [24].

The DN4 test was used in our study to detect neuropathic pain. The mean DN4 score in patients before SPSIPB was 7.76. Neuropathic pain was detected in all 34 patients in the dermatome associated with PHN (DN4 score ≥ 4). Three months after SPSIPB, the DN4 score decreased to 4.26 ± 1.37 (95% CI 3.73–4.68).

The effect of steroid treatment aimed at reducing early-stage sensory nerve damage in PHN is controversial. Jiang et al.‘s meta-analysis found that the effect of oral steroids on preventing PHN during the 6-month follow-up period after the onset of Herpes was uncertain [25]. No patients were using oral steroids in our study. Particulate-free steroids (i.e., dexamethasone) was incorporated into the local anesthetic solution during SPSIPB to prolong the block’s duration and enhance its quality.

Treatment is challenging in patients with chronic pain. In chronic pain, interfascial plane blocks may play an essential role in pain management, particularly by reducing peripheral sensitization [26]. Although there are defined risk factors for PHN, we do not have definitive information about the chronicity process in patients during the acute phase. Park et al. stressed the assistive role of an artificial intelligence-assisted approach in determining the risk and severity of PHN and subsequently establishing appropriate treatment protocols [27]. Recent studies have also suggested that certain predictive factors and biomarkers may be used to determine the risk of PHN in acute Herpes zoster [28]. The protective efficacy of the recombinant zoster vaccine (RZV) has been determined to be 97.2%. The live Herpes zoster vaccine has been shown to reduce the risk of PHN by 66% in individuals aged 60 and older. The US Centers for Disease Control and Prevention (CDC) Advisory Committee on Immunization Practices (ACIP) recommends RZV for individuals aged 50 and older, regardless of previous HZ infection or live HZ vaccination, and recommends 2 doses of RZV or 1 dose of live HZ vaccine for individuals aged 60 and older. RZV is recommended as two doses administered intramuscularly 2–6 months apart, while the live vaccine is recommended as a single dose administered subcutaneously [29]. In our study, none of the patients had received RZV or the live HZ vaccine. Considering HZ complications, we would like to reiterate the importance of highly protective vaccines.

Pain specialists frequently perform radiofrequency ablation of the sensory ganglion of the affected dermatome in PHN. These applications are often performed under C-arm fluoroscopy. PHN frequently occurs in the thoracic region, and epidural applications performed in this region carry risks such as radiation exposure, pneumothorax, nerve damage, bleeding, and infection. Furthermore, epidural procedures in this region are technically more challenging than in other neuroaxial regions, and cutting-edge treatments such as SCS are sometimes used in resistant PHN [5, 30].

The FPBs under ultrasound guidance have been increasingly used for chronic pain in recent years. Since these procedures are performed under ultrasound guidance, there is no radiation exposure. With real-time imaging, the risk of pneumothorax, bleeding, and nerve damage is relatively low. Various FBPs described recently for the management of acute and chronic thoracic pain include ESPB, rhomboid intercostal block, pectoral nerve blocks (PECS I–II), serratus anterior block, retrolaminar block, transversus thoracis plane block, subserratus plane block, parasternal block, and the mid-point transverse process–to-pleura block [26, 31, 32]. FPBs are most frequently used for perioperative analgesia in the setting of acute pain. However, knowledge regarding their use as the primary anesthetic technique in thoracic surgeries—which carry high morbidity and mortality—remains limited. Manici et al. reported successful use of SPSIPB as the sole anesthetic technique in a high-risk patient undergoing video-assisted thoracoscopic surgery (VATS) [33].

In the field of chronic pain, FPBs are applied in conditions like myofascial pain syndrome, post-amputation and post-mastectomy pain, chronic postoperative pain after disc surgery of the lumbar vertebrae, and radiculopathies [34–36].

Substantial evidence indicates that early administration of FPBs during the acute phase of herpes zoster reduces the incidence of PHN. Chuang et al.‘s meta-analysis reported that FPBs with local anesthetics and steroids provided adequate analgesia, reduced analgesic consumption, and decreased the incidence of PHN in patients with acute thoracic AHZ. The authors recommended the ESPB based on its safety profile [37]. Balcı et al. reported that the application of SPSIPB in a patient with acute herpes zoster (AHZ) resulted in a reduction in pain intensity and an improvement in quality of life [38]. Although specific risk factors increase susceptibility to PHN, its occurrence cannot be predicted with certainty. Therefore, in the context of AHZ, the patient’s risk profile and the potential complications of the intervention should be carefully considered through a risk–benefit assessment when evaluating FPBs.

The optimal effective dose of local anesthetic for FPBs is not well defined, and practices vary widely. This variation in practice can increase the potential risk for local anesthetic systemic toxicity. Myotoxicity associated with local anesthetics is another concern, though its true incidence has yet to be determined [39]. We believe that this uncertainty about the optimal anesthetic dose represents one of the significant limitations of FPBs. In our study, we used a total volume of 30 mL, consisting of 0.25% bupivacaine (28 mL) combined with 8 mg (2 mL) of dexamethasone, with no observed procedure-related complications.

SPSIPB is a plane block defined by Tulgar et al. and has been utilized for several years. Although SPSIPB is applied at a single level, studies have demonstrated that it provides analgesia over a broad area in the thoracic region. In a cadaver study by Tulgar and colleagues, dye spread with the SPSIP block was observed in the C7–T7 dermatomes; in clinical practice, sensory block has been reported in the C3–T10 dermatomes [9]. This finding suggests that SPSIPB may have a wider dermatomal distribution than blocks such as the erector spinae plane block (ESPB), paravertebral block (PVB), or rhomboid intercostal plane block (RIB). Previous studies have shown that ESPB and PVB often yield similar analgesic efficacy. Still, PVB can deliver a deeper and more anterior spread, while more superficial techniques, such as the retrolaminar block (RLB), are less invasive and carry a lower risk. Due to these differences, the low complication risk and the reported wide dermatomal spread of SPSIPB may represent potential advantages over ESPB, PVB, and RLB [40–42]. For these reasons, SPSIPB was selected as the second-line treatment after medical therapy. However, SPSIPB remains a newly defined FPB, and there is insufficient evidence to confirm its superiority over existing thoracic plane blocks or its use in place of other FPBs. The preference for SPSIPB prior to epidural procedures is based on the risks associated with thoracic epidural procedures, as previously discussed.

Therefore, SPSIPB has been successfully used for postoperative analgesia in thoracic and breast surgery in anesthesiology in recent years [43, 44]. Since sympathetic block is not expected in the SPSIP block, fewer hemodynamic changes are anticipated. There were no hemodynamic problems with the patients in our study. Considering the spread of SPSIPB, only patients with PHN in the T1-T10 dermatomes in the thoracic region were evaluated in our study. We cannot evaluate the T11-T12 dermatomes of the thorax. In our study, SPSIPB was associated with higher quality-of-life scores in all patients at 1 and 3 months. No procedure-related complications were observed.

Current understanding of the use of FPBs in chronic pain is limited. Future controlled studies are necessary to provide further guidance in this area.

## Conclusion

In this retrospective single-arm cohort, ultrasound-guided SPSIPB was associated with clinically meaningful reductions in pain scores and improvements in quality-of-life measures in patients with thoracic postherpetic neuralgia. However, due to the absence of a control group, non-standardized background pharmacotherapy, and the inherent limitations of retrospective observational design, these findings cannot establish causality or treatment-specific efficacy. The results should therefore be interpreted as exploratory and hypothesis-generating. Well-designed prospective randomized controlled trials with appropriate comparators and standardized pharmacological regimens are necessary to determine the true therapeutic value and comparative effectiveness of SPSIPB in chronic PHN.

### Limitations

This study has several important methodological limitations that must be carefully considered. First, the retrospective, single-center, single-arm design without a control or comparison group precludes any causal inference. The observed improvements in pain intensity and quality of life scores cannot be definitively attributed to SPSIPB. Regression to the mean, placebo effects, spontaneous symptom fluctuation, and the natural course of PHN may have contributed to the observed changes. Second, concomitant neuropathic medications (gabapentinoids, tricyclic antidepressants, and SNRIs) were not standardized, protocolized, or quantitatively adjusted across participants. This lack of treatment uniformity represents a significant confounder and limits attribution of analgesic benefit specifically to the intervention. Third, the magnitude of pain reduction observed in this cohort appears substantial for chronic PHN and should be interpreted cautiously. Without a comparator arm, the durability and true treatment-specific effect size remain uncertain. Fourth, the relatively small sample size limits statistical precision and generalizability. Although exploratory power analysis suggested adequate sample size for within-subject change detection, the study was not powered to evaluate predictors of response or subgroup effects. Fifth, follow-up was limited to three months, and long-term sustainability of analgesia remains unknown. Finally, the retrospective registration of the study protocol introduces the possibility of reporting bias. Accordingly, the findings should be considered exploratory and hypothesis-generating rather than confirmatory evidence of efficacy. 

## Data Availability

The datasets used and/or analyzed during the current study are available from the corresponding author on reasonable request.

## Abbreviations

ACIP: Advisory Committee on Immunization Practices
AHZ: Acute Herpes Zoster
BMI: Body Mass Index
CDC: Centers for Disease Control and Prevention
DN4: Neuropathic Pain Questionnaire 4
ESPB: Erector Spinae Plane Block
FPBs: Fascial plane blocks
HZ: Herpes Zoster
MCS-12: Mental Component Scale-12
NRS-11: Numeric Rating Scale-11
PCS-12: Physical Component Scale-12
PHN: Postherpetic neuralgia
PVB: Paravertebral block
RIB: Rhomboid Intercostal Plane Block
RLB: Retrolaminar Block
RZV: Recombinant Zoster Vaccine
SCS: Spinal Cord Stimulation
SF-12: Short form- 12
SPSIPB: Serratus Posterior Superior Intercostal Plane Block
STROBE: Strengthening the Reporting of Observational Studies in Epidemiology
USG: Ultrasound
